# Should Audits Consider the Care Pathway Model? A New Approach to Benchmarking Real-World Activities

**DOI:** 10.3390/healthcare10091798

**Published:** 2022-09-19

**Authors:** Chun Shing Kwok, David Waters, Thanh Phan, Phyo Kyaw Myint, Gregory Y. H. Lip

**Affiliations:** 1 Department of Post-Qualifying Healthcare Practice, Birmingham City University, City South Campus, Birmingham B15 3TN, UK; 2 Department of Cardiology, University Hospitals of North Midlands NHS Trust, Stoke-on-Trent ST4 6QG, UK; 3 Ageing Clinical and Experimental Research (ACER) Team, Institute of Applied Health Sciences, University of Aberdeen, Aberdeen AB25 2ZD, UK; 4 Liverpool Centre for Cardiovascular Science, University of Liverpool, Liverpool L69 3BX, UK; 5 Aalborg Thrombosis Research Unit, Department of Clinical Medicine, Aalborg University, 9000 Aalborg, Denmark

**Keywords:** clinical audit, pathways, outcomes, quality of healthcare, real-world care

## Abstract

Clinical audit is a method to assess the quality of healthcare services based on whether standards are met or not met. This approach is limited because it fails to recognize how decisions that take place over time and the natural progression of disease has an impact on what happens to patients and the care they receive. The aim of this paper is to introduce the concept of care pathway and explain how care pathways can be audited to better understand care. The care pathway is defined by clinically relevant events that take place within one or more healthcare institutions. The process begins with defining an ideal care pathway which is created by considering local expertise and guidelines. It is then possible to audit against the extent to which this ideal care pathway is achieved. This care pathway audit can enable identification of patterns in real-world care which can help with the of design interventions to help shift patients from the less to more desirable pathways. We conclude that through the process of the care pathway audit cycle, it is possible to learn about real-world activities, better utilize resources, promote safer care, improve quality of care, and help develop more effective interventions.

## 1. Introduction

Clinical governance is the system through which the National Health Service organisations are accountable for continuously improving quality of their services and safeguarding high standards of care by creating an environment in which clinical excellence will flourish [1]. One of the seven pillars of clinical governance is clinical audit [2]. Clinical audit is defined as a quality improvement process that seeks to improve patient care and outcomes through systematic review against explicit criteria and implementation of change [3]. The audit cycle contains five stages, namely, preparing for audit, selecting criteria, measuring performance level, making improvements, and sustaining improvement [4].

An essential part of auditing is defining standards, which is a statement, reached through consensus, that clearly identifies the desired outcome that should be both measurable and achievable [5]. While it is possible to carry out a clinical audit on multiple standards related to the care of a patient with a particular condition, the real-world activities of a patient are complex, and the measuring of multiple individual standards fails to recognise how decisions together with natural progression of disease has an impact on the overall quality of care. The pathway is important as events do not happen in isolation and the pathway approach considers how initial upstream decision making and events may have significant downstream consequences. The general expectation is that not only the correct diagnosis is suspected, tests are undertaken to confirm the diagnosis, and appropriate treatment is started, but also that this sequence of events are undertaken in a timely manner with minimal wastage of resources. This simple model of activities is best described as a care pathway or a sequence of events that are clinically significant events that take place.

## 2. Patient Pathways

**Figure 1:**
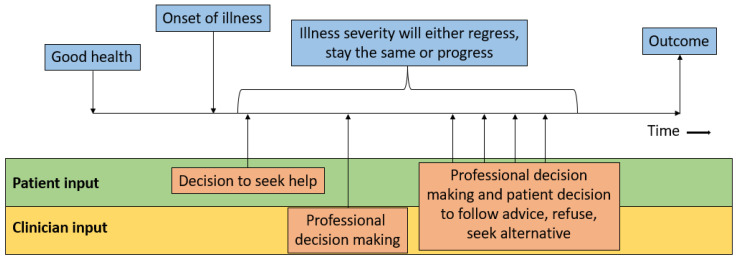
The natural disease progression.

Patient and clinician input into patient and care pathways along the natural progression of disease is outlined in Figure 1. Starting with good health, at some point there is the onset of illness. The disease will progress until the point where the patient decides to seek medical attention. The decision to pursue help is influenced by many factors including the priorities of the patients, severity of the symptoms, and the factors that influence care access including the need to pay for care and the patient’s personal knowledge about health and available services. The priorities of the patient may influence decision making because patients may ignore their symptoms and not seek professional advice because they must work, they have care responsibilities, or they have other engagements or activities. The patient may decide at a particular time point to seek help from a professional, and there is the further decision making regarding who they decide to seek assistance from. It could be a pharmacist, general practitioner/family doctor, other community practitioner, specialist in outpatient settings, or physicians or nurses in the emergency department. They will then be clinically evaluated, and decisions will be made by the clinicians involved, and the advice/treatment suggested may or may not be followed; moreover, the condition may stay the same or progress before an eventual outcome. The clinician decision making refers to the organisation of tests and investigations, referrals to other specialists, and any management they instigate. Real-world possibilities need to be considered, such as the experience and expertise of the practitioner, as misdiagnosis and delay to correct diagnosis are everyday realities.

For example, misdiagnosis has been identified to be frequent in conditions such as acute myocardial infarction [6], heart failure [7], aortic dissection [8], and pulmonary embolism [9]. Moreover, there may be limited resources or services in the area where the patient lives as there may be discrepancy in ease of access to healthcare depending on whether the area is urban or rural. Another important factor is the ability of the patient to pay for healthcare which can impact healthcare-seeking behaviour in countries where healthcare is not free.

**Figure 2:**
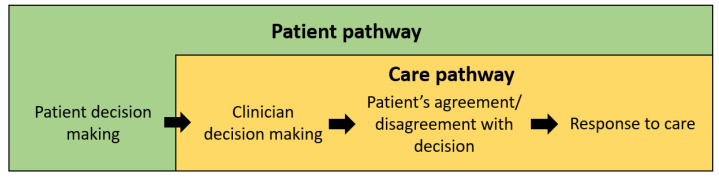
The patient and care pathway.

It becomes apparent that three key factors exist in the model which influences overall outcomes, namely, patient decision making, clinician decision making, and natural disease progression/response to treatment. Considering these factors, two pathways emerge, wherein pathways are defined by relevant events that take place over time that influence the desired outcome. The two pathways are shown in Figure 2. The first pathway is the patient pathway. This considers the initial patient decision making prior to healthcare contact, as well as the care pathways, which outline what happens to patients after they seek healthcare professionals.

In this context, the patient pathway refers to the sequence of clinically relevant events for a patient with a particular symptom, condition, or management. This differs from the patient journey, which is a series of passive events within the health system. The reason why the care pathway needs to be differentiated from the patient pathway is because the care pathway may be more easily influenced by healthcare professionals while the initial steps of the patient pathways is more influenced by public health measures. The upstream events of patient decision making to seek help and who they decide to see is important as some conditions have established treatments that only have a window of opportunity to be of benefit. For example, in the case of acute myocardial infarction, delay to diagnosis could result in patients developing heart failure, and in some of these cases, coronary revascularisation of infarcted myocardium may have limited benefit. Another example may be the case of a patient with lung cancer, where curative treatment may not be an option if there is advanced disease at time of identification and there are only palliative management options.

**Figure 3:**
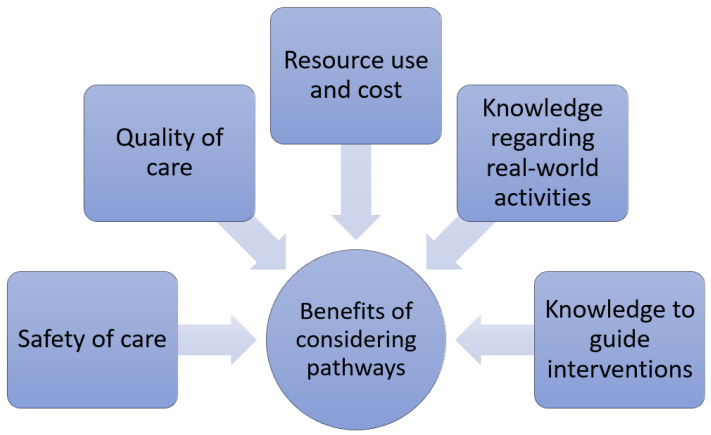
The benefits of considering pathways.

Knowledge of these pathways are important for several reasons (see Figure 3). First, certain paths are associated with better quality of care, while others suggest poor care. Second, there may be paths associated with safer practices compared to higher-risk practices where patients may be exposed to avoidable adverse events. Third, the paths have different resource utilisation and associated cost. Fourth, pathways provide knowledge about real-world activities, and exploring the possibilities may enable understanding of paths which are less apparent but may be of significance. Moreover, it is possible that it may be perceived that there is a significant problem associated with a path that is undesirable, but the real-world data may show that this theoretical problem is infrequent. Finally, the knowledge of the paths may guide interventions which may help shift patients from less favourable to more favourable pathways. This is particularly important if we are to reduce any missed opportunities for better care or more evidence-based practices. Indeed, the need for integrated or holistic approaches to managing many long-term chronic conditions has necessitated the need for multidisciplinary team involvement in patient care pathways [10,11,12].

Take the example of atrial fibrillation, which is the commonest heart rhythm disorder where the management has moved towards appropriate characterisation [13], followed by holistic management addressing avoidance of stroke, patient-centred symptom-directed decisions on rate or rhythm control, and comorbidity and risk factor optimisation [14]. Such an approach has been associated with improved clinical outcomes [15] and is therefore recommended in guidelines [16]. Overall, understanding the paths for patients and care for patients enables exploration of potential missed opportunities to improve the patient experience.

## 3. The Patient Pathway Review

The first method of attempting to understand the possible patient pathway was the patient pathway review [17]. This review was developed to utilise the knowledge from experts to explore what possible events can occur to patients. It has been demonstrated in several conditions, including stable chest pain [18], atrial fibrillation [19], and COVID-19 [20]. The approach of this method, in summary, is defining an ideal pathway based on clinical expertise and evaluating each step of the ideal pathway critically to develop a real-world pathway. A tenant of this type of review is that events do not happen without a reason, and by considering the different perspectives such as the patient, clinician, healthcare service and society, it is possible to attempt to explain the reasons for the paths that take place. The main limitation of this approach is that there is no original data to support the defined paths, and there is no information regarding the relative proportion of patients in each path. The relative proportion of patients in each path is important as this determines the extent or significance of the problem. If the undesirable path is infrequent, it may be accepted that the benefit-to-cost of taking measures to avoid it would not be favourable for the health service.

## 4. Care Pathways

Care pathways differ from patient pathways as highlighted above because they only consider the decision made after contact and advice is sought from healthcare professionals. In addition to clinician’s decisions, care pathways are influenced by the way services deliver care and how they operate. Care pathways can be defined by clinically relevant events that take place within one or more care providers. Considering care pathways for patients within an institution is useful to understand the heterogeneity in patients, care, and outcomes for patients that present with a particular condition. Heterogeneity in patients refers to differences in demographics, comorbid illness, and disease severity at presentation. Heterogeneity in care defines what differences in care are, which could be investigations and treatments for different patients. This also includes patient decision making such as the decision not to have treatment. Heterogeneity in outcomes refers to what possible outcomes might take place such as survival, complications, or death. This has implications in health service design, clinical management algorithm development, knowledge regarding the average and variation in resource use, and the cost of management for specific health conditions.

## 5. Auditing of Care Pathways

**Figure 4:**
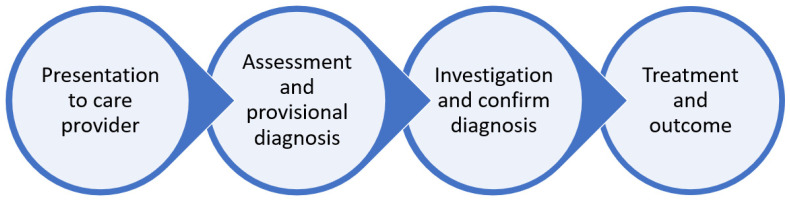
The ideal care pathway.

Considering the model created by the patient pathway review where there is an ideal pathway, it is possible to define an ideal care pathway that can be audited against. In the generic ideal patient pathway, there is an onset of disease, the patient’s decision to seek professional healthcare advice, diagnosis, management, and response. The ideal care pathways would be that the patient presents for help with a condition to an institution, the diagnosis is made, investigations are performed, treatment is started, and the patient has an outcome (Figure 4).

While it appears straightforward, the significance of the pathway may be best illustrated in an example. Consider the example of a patient presenting with chest pain with a diagnosis of NSTEMI. The best source of information to define the ideal care pathway in NSTEMI would be clinical guidelines such as the American Heart Association/American College of Cardiology guidelines [21], European Society of Cardiology guidelines [22], and the National Institute for Clinical Excellence guidelines in the United Kingdom [23]. Considering these together with local practice and desires for clinical practice, it would not be unreasonable to expect the following standards for an audit: (1) all patients are identified within 24 h of admission; (2) all patients have at least 24 h of cardiac monitoring, (3) all patients are considered for a coronary angiography within 72 h, (4) all patients are considered for antiplatelet and secondary prevention medications, (5) all patients have an inpatient echocardiogram, and (6) all patients are discharged within 5 days of admission. Considering these individual standards, one finds it is possible that the ideal care pathway is that the patient admitted with symptoms potentially related to NSTEMI should ideally have the diagnosis confirmed within 24 h, and then the patient has at least 24 h of cardiac monitoring, and following this, the patient is considered for coronary angiogram, the patient should have treatment with medical acute coronary syndrome treatments, the patient has an inpatient echocardiogram, and the patient is discharged within 5 days.

The reason why the pathway should be audited against rather than individual standards is important for a few reasons. First, it is useful to know how many patients received optimal care. Second, it is possible that the standards are linked such that the same patient who does not have a diagnosis within 24 h is the same patient who does not have cardiac monitoring and has a prolonged length of stay. An example of why this might occur is a patient being initially misdiagnosed with pneumonia, acute heart failure, or pulmonary embolism that was having an NSTEMI, with the delayed diagnosis potentially contributing to the failure to achieve multiple standards. Third, considering the care pathways provides insight as to what occurs in terms of relevant events over time in real-world settings. This can help determine the resource use and average cost in relation to overall outcome and standard achievement for individual patients. This information would be helpful for service design, care algorithm development, and the creation of other interventions.

The data collection and analysis in the audit aims to determine what care pathways exist, and this is a data-driven approach. Patterns can be observed based on the frequency of variables collected and how these variables may or may not be associated with other variables. An example of the association between variables is the case where a patient undergoes an operation which carries a risk of a complication. If the patient does not have the operation, they will not have the complication, so the complication is associated with the procedure. This is important as every decision made in the care of a patient can have consequences as investigation and treatment may carry risks of adverse events and have downstream consequences including delay to optimal diagnostic test and effective treatment. The variables collected depend on the exact area where the auditing is taking place, but they may include the healthcare professionals who cared for the patient, decisions made regarding investigations and management, and patient and healthcare outcomes. It is important that not only a variable is collected but also the sequence in which it occurred over time. This is relevant with the growth of research which analyses databases, as some databases may lack the granular detail in data that enables these types of evaluations. The data collected can then be used to determine sequences of clinically relevant care-related events and outcomes for a patient, and when multiple patient journeys are analysed in a similar way, the care pathways can be determined. This information regarding what care paths patients may take can be used by relevant stakeholders to determine what is more desirable, acceptable, and undesirable in the benchmarking process. In addition, it can help to determine if practice needs to change because a problem is significant but also it provides detailed knowledge that can be helpful in the intervention development process which may aim to shift patients from less to more desirable care pathways.

## 6. The Care Pathway Audit Cycle

**Figure 5:**
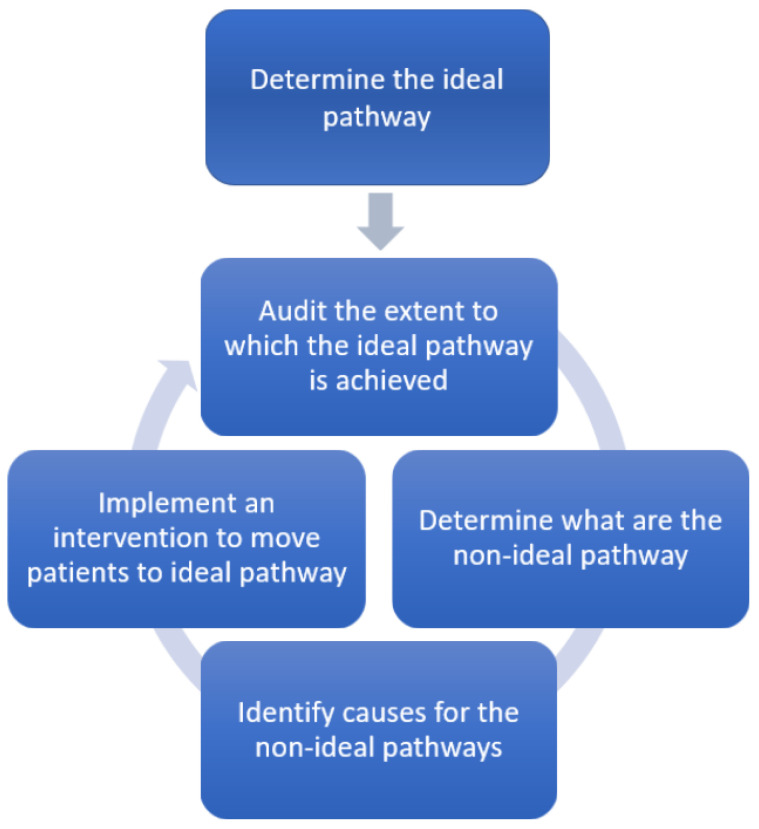
The care pathway audit cycle.

The basic steps of an audit cycle include identifying a problem, defining the standards or criteria, collecting data, analysis, implementation of change, and re-auditing [24]. Rather than having standards or criteria, the audit can be carried out against the ideal care pathway. The ideal care pathway should be based on local expertise together with guidelines. This is important as it is not always possible that guideline endorsing management are implemented in all places such as rural areas where resources may be scarce. In this new model (Figure 5), the starting point is defining the ideal care pathway to audit against, audit the extent to which the ideal pathway is achieved, determining the extent to which non-ideal pathways occur, and defining what might be the cause of non-ideal pathways and implementation of an intervention to move patients to the ideal pathway. The auditing involves all the steps of data collection and analysis.

A proposed framework of steps to carry out a care pathway audit is shown in Table S1. The key areas of consideration in this framework are as follows: (1) designing the evaluation and pilot evaluation, (2) seeking approval, (3) conducting the care pathway audit and interpretation of the findings, (4) dissemination of findings, and (5) considering intervention and re-auditing.

An important consideration is determining what the non-ideal pathways are and how frequently they occur. In the example of NSTEMI, cardiologists may have ideas as to why patients do not meet the ideal care pathway, but they may not actually know what happens in real-world settings. Suppose the interest is in reducing paths where there are delays to identification and treatment which contribute to prolonged hospital stay. While most cardiologists who review a patient with chest pain would do the simple tests to exclude acute myocardial infarction, not all patients present with chest pain and not all clinicians will think the same way about chest pain. Many professionals may be involved in the care of patients who present acutely to hospital such as nurse practitioners carrying out triage of patients, emergency department doctors, and acute medical physicians. It is possible that these professionals may make the wrong diagnosis, as acute myocardial infarction may be mistaken for nonspecific chest pain, gastrointestinal disease, musculoskeletal pain, and arrhythmias [6]. Furthermore, acute myocardial infarction may present without chest pain [25], and some cases may be challenging to diagnose early as the patient has atypical symptoms. The extent to which this occurs is specific to the local healthcare setting, and it may be that this is a problem in one centre but not another. The care pathway audit is necessary because data are needed to determine if there is indeed a problem with the frequency of patients who go through a non-ideal pathway. A key consideration is data that need to be collected to understand what occurs in real practices as it is possible that there may be unforeseen reasons for certain paths to take place which are only found through data collection.

Once the non-ideal care pathways are defined, the next important consideration is the reasons why they occur. This is complex and may involve the consideration of different perspectives including the patient, clinician, health service, and society and is best substantiated with data which can be collected during the auditing process. In the example of the NSTEMI, it is possible that the patient was on target to achieve the ideal pathway having had the diagnosis, monitoring, medical therapy, and revascularisation treatment but had to stay in hospital for beyond 5 days because they had to wait for an inpatient echocardiogram which took several days to arrange. This is important because the cause of the deviation from the ideal pathway is a result of the need to wait for the inpatient echocardiogram, which is different from a patient who had to stay beyond 5 days because they had to wait for a coronary angiogram because they were admitted at a time when there were many patients who required emergency treatment.

## 7. Discussion

This report introduces the concept of the care pathway and explains how care pathways can be audited to better understand care. This method is an alternative to the traditional clinical audit which evaluates the extent to which standards are met. The care pathway approach places individual audited events within a framework of events over time where upstream events can have downstream consequences. In addition, taking a real-world data-driven care pathway approach enables defining the non-ideal pathways that take place that may have not been recognised by researchers. By determining both what non-ideal pathways occur and how frequently they occur can help determine if there is a potential opportunity for care to be improved. The information determined from the care pathway audit can be utilised to justify the investment of resources or changes in care practices to enable interventions to optimise the quality of care delivered, promote safer practices, reduce unwanted variations in services, and reduce unnecessary costs.

The care pathway audit has the advantage that the information has use in the setting where data are collected. Healthcare is different depending on whether care takes place in the community or hospital settings, in urban or rural areas, and in private compared to publicly funded healthcare systems. As resources are limited, health services are set up by one or a group of individuals to meet the healthcare requirements of the area. The reality is that timely access to investigation and treatment is not available at all centres. Therefore, the ideal pathway may differ depending on the setting. For example, in a rural setting, the goal may not be to have patients who are identified to have acute ST-elevation myocardial infarction to receive primary PCI but to receive timely fibrinolytic therapy. The goal may also be for patients to be rapidly identified and transferred to another unit which is capable of primary PCI within a certain timeframe. Even in two rural centres, there may be physical geographical differences which influence time to definitive treatment together with issues related to transport methods, whether by plane, helicopter, or by ambulance. By defining the care pathways, one can better understand the extent to which locally expected care occurs or does not occur. If a problem is found with a high prevalence of undesirable pathways for patients, it is possible to intervene to try to shift patients to more favourable care pathways.

A basic premise in setting up a health service is that those who deliver care should be aware of what care they aim to deliver. This is particularly important for healthcare service managers who employ staff and define care protocols and management guidelines. By considering different stakeholders who are involved in the process, it should therefore be possible to define what objectives there are that should be achieved over the course of time in an ideal care pathway.

The approach of collecting data to define pathways for patients from real-world settings generate high-quality data because there is less proneness to assumptions. For most data collection, the decision on what to collect is based on one or more expert opinions or with reference to the literature. In the case of the care pathway, audit data are collected for multiple patients, and the process is data driven, which enables discovery or capture of unsuspected pathways. The data-driven data collection can be partially supplemented by considering what is known in the area in terms of existing research and published literature in terms of variables to collect. This is important as a key principle is that if the data are not collected, their significance in affecting associations with other variables and outcomes will never be determined. Because discharge letters are more concise, they are often chosen to as a source of data rather than notes, and there may be assumptions made about the quality of the data. It is often assumed that the material available in discharge letters is satisfactory, but there is no guarantee that important information that is present in the patient medical records is not omitted. For example, a discharge letter may state that a patient came to hospital for NSTEMI and heart failure and received medical treatment and coronary revascularisation before being discharged 10 days later. All the facts in the discharge letter may be correct, but the letter may fail to say that the patient was admitted and initially treated for a chest infection and sepsis because they had shortness of breath and chest pain. However, when fluid was administered, they went into acute pulmonary oedema and their prolonged length of stay was because the initial diagnosis was not correct, and a period was needed to provide diuresis before the patient could safely lie flat for their coronary angiogram and percutaneous coronary intervention.

The care pathway audit has two primary goals. The first goal is to define to what extent real-world practices reflect what is expected. The second goal is to define why the non-ideal pathways occur. The second goal requires a degree of flexibility in the data collection as these paths may not be foreseen in the initial planning of the study. Furthermore, there may need to be a consideration of capturing data regarding factors that may explain why these undesirable pathways may occur. This consideration of factors that may influence deviation from the ideal pathway is important because missed opportunities to have more desirable care pathways may be classified as avoidable or unavoidable. For instance, there could be a patient that refuses treatment that is offered. In example of NSTEMI, a patient has a right to decline the invasive procedures. This would be an unavoidable missed opportunity if the decision that was made by the patient was an informed decision where the risk and benefits of the procedure was explained, and the patient freely made the decision not to have this management. However, if information was presented to them in a way that made them feel that the procedure was at greater risk of harm then benefit, then it would be a missed opportunity, especially if there were downstream consequences such as readmission for heart failure.

One of the tenants of the patient pathway review is that events do not occur without a reason. Therefore, a systematic approach of considering different perspectives of those involved in the pathway process can help to understand the context within which observed events occur. These perspectives include the patient, clinician, healthcare service, and society’s perspective. The major difference between the patient and the care pathways is that the patient pathway is centred on the patient and what happens to them in terms of decision making regarding their health as they transition to the care of one or more professionals in the community or hospital settings. The care pathway, on the other hand, may be focused on the interactions of a patient within one or more institutions. Therefore, the healthcare providers and their policies for clinical practice plays a more significant role in influencing the care pathway. The types of interventions that can be implemented in the care pathway are easier as they only need to be performed within the environment of the institution. An example of such an intervention would be the development and implementation of a hospital-based rapid access chest pain clinic service [26]. Patient satisfaction and quality of life are important factors to be considered when evaluating the effectiveness of healthcare. While the concept of auditing practice is centred around determining if standards are met or not met, the perspectives of the patients should be acknowledged. In auditing clinical pathways, one can determine the extent to which the clinician determined ideal pathway is met but the defined pathways in this process can also be evaluated from the patient perspective in terms of favourable compared to less favourable pathways. There are interests regarding the ideal pathway of care that healthcare services and patients share such as the need to minimise delay to care; minimise misdiagnosis; and striving for pathways which promote the best outcome in terms of patient survival, lower morbidity, and better quality of life. However, in public healthcare systems where there are no additional charges to patients associated with the care delivered, the services may also be interested in reducing cost and minimising unnecessary use of resources compared to the perspective of the patient. In fee-for-service healthcare systems, it is also possible that additional testing may take place that is not necessary as this may generate profit which may not be of interest to patients who must pay for care. There may be further differences in opinion of patients regarding satisfaction depending on how services are set up. For example, patient management can be led by a general physician, a specialist nurse, or a specialist consultant, and there may be variation in a patient’s perception about care quality. There may be additional differences in ongoing care for patients once discharged from services, and this depends on the local availability of follow-up care which can affect quality of life, particularly with availability of services such as community rehabilitation. Therefore, it is important that the audited pathways be considered in terms of different stakeholders including the patient as well as the healthcare service.

Determining the care pathways that patients undergo can be useful in benchmarking. Clinical benchmarking is the systematic process in which current practice and care are compared to, and amended to attain, best practice and care [27]. Central in this process is having a clear understanding of real-world care and the inherent heterogeneity in terms of the patient journey despite guidelines on best practices. By defining what the ideal care is based on service providers and their locally available resources, one can then determine the extent to which this is achieved or not achieved. This can be conducted in a standard clinical audit. However, to appreciate how upstream events can have major downstream consequences, together with what types of paths, some of which may be unforeseen, may occur, the pathway concept was developed. The amalgamation of the pathway concept and auditing has not been conducted before and combining both ideas enable further insight into patterns that yield desirable care and patterns that ultimately yield care that results in poor outcomes. Judgements can further be made by stakeholders regarding whether there were missed opportunities for better care or whether what happened to patients was avoidable or not avoidable. This will enable better decision making in terms of changes to practice as there is additional information not only about the extent to which audited standards are achieved but also what causes both the desired and less desirable outcomes, which is useful information in the design of care quality improvement interventions.

It is important to recognise that clinical governance is not the only system for quality improvement, as most countries such as the United States and Australia have agencies or commissions which help oversee these processes [28,29]. Nevertheless, there may be similarities in the practical approaches of healthcare quality improvement that are shared with the clinical auditing process that is defined within clinical governance such as the need for data collection and analysis to understand care to determine if improvements need to be made.

There are additional challenges associated with data collection for care pathways compared to other observational research methods. The findings in a care pathway review are partly data driven as opposed to evaluations where the collected is entirely pre-defined prior to data collection. This process of acquiring data and attempting to understand why the data was as it was found requires some skill and basic knowledge of the area by the data collector. It may also be more resource intensive and time consuming to review patient or hospital records to find out what occurs from a care perspective to patients as opposed to simply collating the information that is present on discharge summaries. The main limitation of the care pathway is that it is retrospective and relies on the quality of written records. High-quality data and routinely collected data may not be sufficient to conduct this type of pathway audit. For example, hospital discharge summaries will describe what diagnoses and tests were performed during a patient’s inpatient stay. However, there may not be sufficient detail to explain how these events took place over time. For example, a patient discharge summary may say they had three diagnoses acute myocardial infarction, ischemic stroke, and gastrointestinal bleeding and they may have undergone percutaneous coronary intervention. While the stenting is likely to have taken place after the acute myocardial infarction, it may not be known as to whether the gastrointestinal bleeding took place before or after the acute myocardial infarction and before or after the stroke. Similarly, we do not know if the stroke occurred before or after the acute myocardial infarction. This is important as the stroke may be a complication of the coronary stenting procedure and the gastrointestinal bleeding a consequence antithrombotic medication used in the management of both acute myocardial infarction and stroke. Another key limitation is that the approach does not have the perspective of the patient, as this type of evaluation will require consent for the patient to take part in the evaluation and provide their reasoning for their decision making. Moreover, this area of work is that the literature is largely dominated by work from cardiology, and the only study outside of cardiology has been on COVID-19 [20]. More literature is needed from the non-cardiology perspectives on care and patient pathways. In addition, there are potential challenges in that defining pathways may not always enable clear benchmarking for practice and performance as there may be different perspectives depending on the stakeholders. It can further be challenging in some cases to directly correlate observed care pathways and actual clinical governance.

## 8. Conclusions

In conclusion, we describe the concept of the care pathway, which is a sequence of clinically relevant events that take place within one or more healthcare providers. Considering care pathways is important as it can be audited against a pre-defined locally specific ideal care pathway for a particular condition. This is important as it can help define the extent to which ideal care is delivered and if there are any care pathways that patients undergo which are undesirable. By understanding whether there is a problem with these undesirable pathways, together with possible reasons for them, it is possible to design interventions that can help shift patients from the less desirable to more desirable pathways. Through this process of the care pathway audit cycle, it is possible to learn about real-world activities, better utilise resources, promote safer care, improve quality of care, and help develop more effective interventions.

## Data Availability

Not applicable.

## Supplementary Materials

The following supporting information can be downloaded at https://www.mdpi.com/article/10.3390/healthcare10091798/s1, Table S1: Proposed framework of steps to carry out a care pathway audit.
