# Supplementary material for: Should Audits Consider the Care Pathway Model? A New Approach to Benchmarking Real-World Activities
Source: Healthcare (Basel). 2022 Sep 19;10(9):1798. doi: 10.3390/healthcare10091798 (PMC9498525; doi:10.3390/healthcare10091798)
Supplement: Supplementary file 1 [file healthcare-10-01798-s001.zip › healthcare-1872610-SI.pdf]

Table S1. Proposed framework of steps to carry out a care pathway audit

**Proposed framework of steps to carry out a care pathway audit**	
**Designing the evaluation and pilot evaluation**	Define health condition/disease area of interest and setting.
	Define locally/regionally/nationally among experts involved in the care processes that should ideally happen to patients with the health condition/disease of interest.
	Consider a pilot evaluation of local data (e.g. clinical notes, electronic records, etc) to define the data that needs to be collected to define the care pathway or what happens to patients from presentation to discharge/death.
	Define protocol and data collection proforma for the care pathway audit.
**Seeking approvals**	Seek appropriate institutional approvals for audit to take place
**Conducting the care pathway audit and interpretation of the findings**	Conduct the audit against the ideal care pathway defined as per protocol.
	Define to what extent that ideal care pathway is met.
	Define the prevalence of pathways which deviate from the ideal care.
	Review care processes in order to make inferences regarding reasons for deviation from ideal care.
**Dissemination of the findings**	Present findings locally/regionally/nationally to disseminate care so that others can learn from local experience.
**Consider intervention and re-auditing**	Determine if intervention is needed to promote more favourable pathways for patients.
	Considering re-auditing the care pathway after intervening.
